# GSK3β Inhibition by Phosphorylation at Ser^389^ Controls Neuroinflammation

**DOI:** 10.3390/ijms24010337

**Published:** 2022-12-25

**Authors:** Belen Calvo, Miriam Fernandez, Mercedes Rincon, Pedro Tranque

**Affiliations:** 1 Research Institute for Neurological Disabilities (IDINE), Albacete Medical School, University of Castilla-La Mancha (UCLM), 02008 Albacete, Spain; 2 Department of Immunology and Microbiology, University of Colorado Denver, Aurora, CO 80045, USA

**Keywords:** neuroinflammation, microglia, astrocytes, neutrophils, NF-κB signaling, flow cytometry

## Abstract

The inhibition of Glycogen Synthase Kinase 3 β (GSK3β) by Ser^9^ phosphorylation affects many physiological processes, including the immune response. However, the consequences of GSK3β inhibition by alternative Ser^389^ phosphorylation remain poorly characterized. Here we have examined neuroinflammation in GSK3β Ser^389^ knock-in (KI) mice, in which the phosphorylation of Ser^389^ GSK3β is impaired. The number of activated microglia/infiltrated macrophages, astrocytes, and infiltrated neutrophils was significantly higher in these animals compared to C57BL/6J wild-type (WT) counterparts, which suggests that the failure to inactivate GSK3β by Ser^389^ phosphorylation results in sustained low-grade neuroinflammation. Moreover, glial cell activation and brain infiltration of immune cells in response to lipopolysaccharide (LPS) failed in GSK3β Ser^389^ KI mice. Such effects were brain-specific, as peripheral immunity was not similarly affected. Additionally, phosphorylation of the IkB kinase complex (IKK) in response to LPS failed in GSK3β Ser^389^ KI mice, while STAT3 phosphorylation was fully conserved, suggesting that the NF-κB signaling pathway is specifically affected by this GSK3β regulatory pathway. Overall, our findings indicate that GSK3β inactivation by Ser^389^ phosphorylation controls the brain inflammatory response, raising the need to evaluate its role in the progression of neuroinflammatory pathologies.

## 1. Introduction

Glycogen Synthase Kinase 3 (GSK3) is a serine-threonine kinase with broad specificity. GSK3 regulates processes as diverse as inflammation, cell growth, cell differentiation, and energy metabolism and is implicated in age-related diseases such as diabetes, Alzheimer’s disease, and cancer [1]. Both GSK3 isoforms, GSK3α and GSK3β, are ubiquitously expressed, but their functions are not fully overlapping [2].

GSK3β is constitutively active, although its activity can be inhibited by sequestration into cytosolic complexes as well as by phosphorylation at residues Ser^9^ and Ser^389^ [3]. Akt-induced phosphorylation of GSK3β at Ser^9^ is a GSK3β regulatory pathway present in most cell types, and it is the default pathway to limit GSK3β activation and prevent cell death. In contrast, we have found that p38 MAPK-induced Ser^389^ phosphorylation is restricted to specific tissues such as the brain, thymus, and spleen [4] and that brain expression of phospho-Ser^389^ GSK3β is developmentally regulated [5]. The phosphorylation of GSK3β by Ser^389^ is triggered primarily by DNA double-strand breaks response and it is critical to allow DNA repair [6,7]. Thus, while phosphorylation on Ser^9^ is the main pathway for the inactivation of cytoplasmic GSK3β, Ser^389^ phosphorylation is used to inactivate the nuclear pool of GSK3β to increase survival during DNA DSB repair [8]. Failure to inactivate GSK3β through Ser^389^ phosphorylation in response to DSB leads to increased cell death by necroptosis [6,7]. This is highly relevant because the necroptotic type of death is more inflammatory and has been found in inflammatory diseases [9]. However, unlike the phosphorylation of Ser^9^, the implications of phosphorylation of GSK3β on Ser^389^ remain poorly characterized.

A number of studies have supported the association between GSK3 and inflammation. Thus, activation of GSK3 by inflammatory stimuli such as stress, infection, or trauma promotes the production of pro-inflammatory cytokines such as IL-6, IL-1β, and IFNγ, while GSK3β inhibition suppresses the synthesis of pro-inflammatory mediators and increases IL-10 production by immune cells [10]. GSK3β can be activated by lipopolysaccharide (LPS), one of the main immunostimulatory endotoxins of Gram-negative bacteria [11,12]. Upon binding of bacterial LPS to Toll-like receptors (TLRs), GSK3β mediates the release of inflammatory cytokines in cells of the innate immune system [13]. Moreover, it has been reported that the administration of GSK3β inhibitors to mice treated with a lethal dose of LPS significantly increases animal survival [14]. Despite these pro-inflammatory effects associated with GSK3β, anti-inflammatory effects have also been documented [15,16]. Thus, GSK3β regulation is critical to maintaining the balance between pro- and anti-inflammatory cytokines following TLR activation [17]. 

Nuclear factor kB (NF-kB) mediates the production of pro-inflammatory cytokines and is one of the main transcription factors activated by TLRs. Several authors have confirmed that GSK3 is able to activate NF-kB signaling through phosphorylation of the IkB kinase complex (IKK), which phosphorylates IkB and releases active NF-kB. Another transcription factor phosphorylated by GSK3 is a signal transducer and activator of transcription-3 (STAT3), which mediates neuroinflammation in the context of septic shock [18]. STAT3 phosphorylation by GSK3 results in nuclear translocation and activation. In addition, GSK3 inhibits anti-inflammatory CREB and AP-1 transcriptional activities, amplifying the expression of proinflammatory cytokines [10,19]. 

Microglia cells are resident macrophages in the CNS. Together with infiltrated leukocytes, microglia activation plays a critical role in neuroinflammation. Astrocytes are also glial cells with neuroimmune functions as they crosstalk with microglia, leukocytes, and endothelial cells, thus modulating both innate and adaptive immune responses [20]. Activation of microglia and astrocytes in response to inflammatory stimuli (gliosis) is characterized by major cellular changes that include proliferation, hypertrophy, and cytokine release [21]. GSK3β has been involved in the development of gliosis in response to LPS or by overexpression of neural GSK3β [22]. There is also evidence that glial reactivity and neuroinflammation induced by GSK3β can be mediated by activation of the NF-kB pathway in the brain [19,23]. In contrast, the inactivation of GSK3β decreases glial reactivity, restricts the neuroinflammatory response, and increases anti-inflammatory CREB activity in neural cells [24]. Consequently, it has been suggested that GSK3β plays a fundamental role in the progression of neuroinflammatory disorders, such as Alzheimer’s disease and multiple sclerosis [25]. 

Phosphorylation is a regulatory mechanism of GSK3 activity with major implications for the control of the immune response. Thus, GSK3 inhibition by classical Ser^9^ phosphorylation upregulates the production of anti-inflammatory mediators and downregulates the release of pro-inflammatory cytokines [26], although it is also involved in the resolution of inflammation in a model of experimental colitis induced by trinitrobenzene sulfonic acid [27]. Within the brain, defective inhibition of GSK3β by Ser^9^ phosphorylation has been related to inflammatory neuropathologies such as Parkinson’s disease [28] and schizophrenia [29]. In contrast, despite the high levels of phospho-Ser^389^ GSK3β found in the brain [4], it remains unknown whether this pathway for inactivation of GSK3β plays a role in tuning neuroinflammation. 

Here, we investigate the possible role of Ser^389^ phosphorylation-mediated GSK3β inactivation in neuroinflammation using GSK3β KI mice in which GSK3β Ser^389^ was replaced by Ala to prevent the C-terminal phosphorylation of GSK3β [7,8]. Our results reveal that failure to inactive GSK3β by phosphorylation at Ser^389^ under physiological conditions leads to chronic low-grade neuroinflammation as well as the lack of response to inflammatory stimuli such as LPS. This chronic inflammation seems to be specific to the brain since the peripheral immune system was largely unaffected. We also show that the absence of phospho-Ser^389^ GSK3β in the brain of GSK3β KI mice regulates NF-kB signaling, which is a central event in the control of neuroinflammation. Therefore, our findings provide support for the involvement of GSK3β inactivation by Ser^389^ phosphorylation in inflammatory neuropathologies. 

## 2. Results

### 2.1. Failure to Inactivate GSK3β by Ser^389^ Phosphorylation Leads to Increased Basal Neuroinflammation

Inactivation of GSK3β by phosphorylation on Ser^9^ has been associated with an anti-inflammatory stage in the brain [30]. Although the inactivation of GSK3β by phosphorylation on Ser^389^ is important to reduce neuronal death caused by DNA double-strand breaks in the brain, no studies have investigated whether this pathway could also regulate the neuroinflammatory response. We, therefore, used the previously described GSK3β Ser^389^ KI mice, in which Ser^389^ is replaced by Ala, to prevent Ser^389^ phosphorylation. We examined resident and infiltrated immune cells in the whole brain (except cerebellum and olfactory bulbs) from WT and GSK3β Ser^389^ KI mice by mechanical dissociation of the tissue and Percoll gradient, following a previously optimized protocol to obtain a balanced mixture of resident and infiltrated cells in the brain, including microglia/macrophages, astrocytes, infiltrated neutrophils and lymphocytes [31]. Cells were then analyzed by flow cytometry using standard markers (Figure S1 in Supplementary Materials) [31,32,33]. Activated and resting microglia/macrophages were identified as CD11b^+^CD45^high^ and CD11b^+^CD45^low^, respectively, whereas the lymphocytic population was marked as CD45^+^CD11b^−^ [32,34]. Neutrophils were characterized as CD45^+^Ly6G^+^ cells; and astrocytes were defined within the CD45^−^ population by expression of the cell surface antigen-2 (ACSA-2). This marker is used for the detection of developing and adult astrocytes [35]. Upregulation of ACSA-2 has been observed in reactive astrocytes associated with inflammatory conditions, such as autoimmune demyelination [36]. We previously found that the number of ACSA-2^+^ astrocytes in mice brains increases after LPS treatment, further indicating that the level of ACSA-2 expression correlates with astrocyte activation in response to inflammatory stimuli [31]. Although ACSA-2 can also be expressed in a small subpopulation of microglia and macrophages [35], these cells are within the CD45^+^ population.

**Figure 1:**
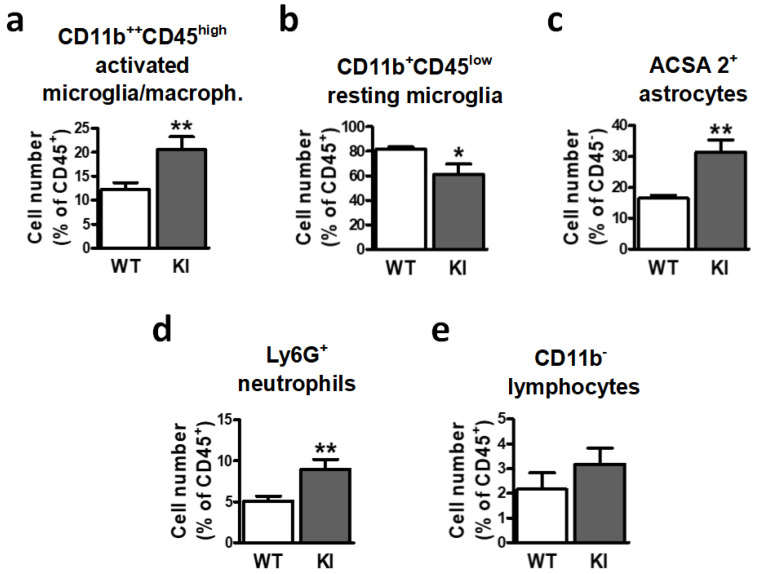
Inactivation of GSK3β by Ser^389^ phosphorylation affects brain cell populations implicated in neuroinflammation. Brains from both WT and GSK3β Ser^389^ KI mice were removed and processed for flow cytometry analysis of (**a**) CD11b^++^CD45^high^ activated microglia/macrophages, (**b**) CD11b^++^CD45^low^ resting microglia, (**c**) ACSA-2^+^ astrocytes, (**d**) Ly6G^+^ neutrophils, and (**e**) CD11b^−^ lymphocytes. Histograms represent cell number, expressed as the percentage of CD45^−^ cells (astrocytes) or CD45^+^ cells (all other populations). Data are shown as mean ± S.E.M (*n* = 7–9). Student *t*-test was applied to compare genotypes. *, *p* < 0.05; **, *p* < 0.01. WT, wild-type; KI, and GSK3β Ser^389^ knockin.

Flow cytometry analysis of the brain cell isolates from mice under physiological conditions revealed a marked increase in the fraction of activated microglia/macrophages in GSK3β Ser^389^ KI mice compared to WT mice (Figure 1a). In contrast, the percentage of resting microglia decreased in the brains of GSK3β Ser^389^ KI mice (Figure 1b). In addition, there was a marked increase in the presence of ACSA-2^+^ astrocytes (i.e., activated astrocytes) in GSK3β Ser^389^ KI mice (Figure 1c). Regarding immune cell infiltration in the brain, there was a clear accumulation of neutrophils in the brain of GSK3β Ser^389^ KI mice relative to WT mice (Figure 1d), further demonstrating the presence of ongoing inflammatory response in the brain of GSK3β Ser^389^ KI mice under physiological conditions. However, the frequency of CD45^+^CD11b^−^ leukocytes (predominantly lymphocytic cells) in the brain was not affected in GSK3β Ser^389^ KI mice (Figure 1e). Together, these results show that failure to inhibit GSK3β by phosphorylation at Ser^389^ leads to a chronic innate inflammatory response in the brain that causes reactivity between microglia and astrocytes.

### 2.2. Lack of Effect of GSK3β Phosphorylation at Ser^389^ on Spleen Cells

To explore whether the altered inflammatory state detected in GSK3β Ser^389^ KI mice is restricted to the brain or whether the effect is also systemic, we performed flow cytometry analysis for the different cell populations in the spleen. Neutrophils were identified as Ly6G^+^ cells. Ly6G expression was highly variable since it depends on the maturation stage of cells, as previously shown [37]. In addition, monocytic myeloid cells were labeled as Ly6G^–^CD38^+^CD11b^+^ cells. These monocytes/macrophages presented two clearly distinct populations based on the levels of CD38 expression, as previously reported [38]. CD8^+^ cells, CD4^+^ cells, and B220^+^ cells (B cells) were also evaluated (Figure S2 in Supplementary Materials).

**Figure 2:**
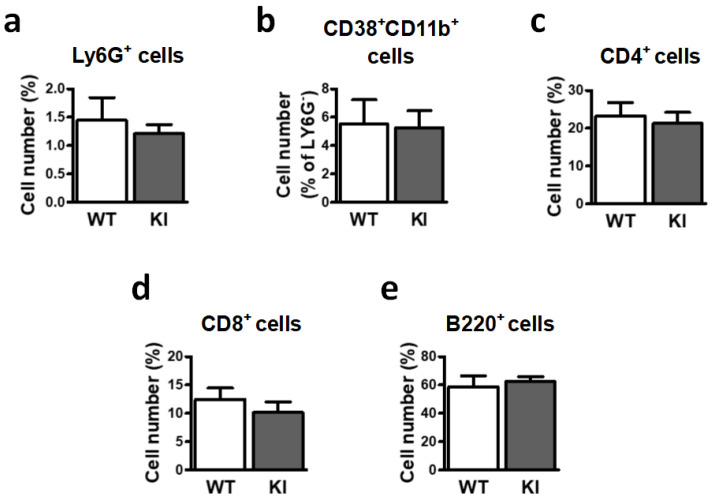
Flow cytometry analysis shows similar spleen cell populations in WT and GSK3β Ser^389^ KI mice. After dissociation of spleen cells, flow cytometry was used to assess the number of (**a)** Ly6G^+^ neutrophils, (**b**) CD38^+^CD11b^+^ monocytes/macrophages, (**c**) CD4^+^ cells, (**d**) CD8^+^ cells, and (**e**) B220^+^ cells. Histograms show cell numbers expressed as percentage of total cells. Data correspond to mean ± S.E.M. (*n* = 5–7). Student *t*-test was applied to compare genotypes. WT, wild-type; KI, GSK3β Ser^389^ knockin.

Comparative analysis of these populations in spleen from WT and GSK3β Ser^389^ KI mice showed no significant differences in the immune cell profiles between both mouse strains under physiological conditions (Figure 2). Thus, the inflammatory stage in GSK3β Ser^389^ KI mice seems to be restricted to the brain, in accordance with the selective accumulation of GSK3β phosphorylation at Ser^389^ in this tissue [4].

### 2.3. LPS Is Unable to Increase Neuroinflammation in GSK3β Ser^389^ KI Mice

**Figure 3:**
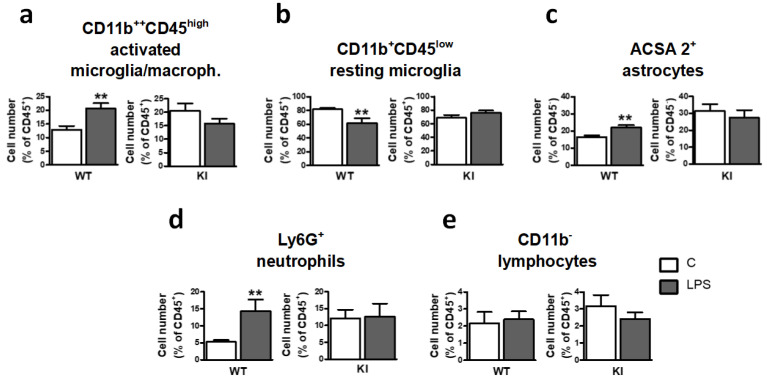
Neuroimmune cells from GSK3β Ser^389^ KI mice fail to respond to LPS. Adult WT and GSK3β Ser^389^ KI mice were injected intraperitoneally with LPS (0.5 mg/kg). Three days later, brain populations of (**a**) CD11b^++^CD45^high^ activated microglia/macrophages, (**b**) CD11b^+^CD45^low^ resting microglia, (**c**) ACSA-2^+^ astrocytes, (**d**) Ly6G^+^ neutrophils, and (**e**) CD11b^−^ lymphocytes were evaluated by flow cytometry. Histograms show LPS effects on cell number in both genotypes. Data are presented as percentages of CD45^−^ cells (astrocytes) or CD45^+^ cells (all other populations), and values correspond to mean ± S.E.M (*n* = 7–9). Student *t*-test was applied to evaluate the significance of changes induced by LPS in each genotype. ** *p* < 0.01. WT, wild-type; KI, GSK3β Ser^389^ knockin; LPS, lipopolysaccharide.

Next, we investigated whether abolishing Ser^389^ phosphorylation, besides causing basal tonic inflammation, could also affect the inflammatory response triggered by LPS. WT and GSK3β Ser^389^ KI mice were injected intraperitoneally with a sublethal dose (0.5 mg/kg) of bacterial lipopolysaccharide (LPS). The brain inflammatory response was examined three days later since such treatment has been previously shown to produce low-grade inflammation in mice brains [39,40]. This LPS response also resembles the chronic inflammatory state observed in several neurological diseases, including depression, anxiety, and Parkinson’s disease [41,42]. As expected, LPS induced glial reactivity in WT mice, as determined by the increase in the frequency of activated microglia/macrophages (Figure 3a) and decreased frequency of resting microglia (Figure 3b). In contrast, LPS failed to trigger any further inflammatory response in GSK3β Ser^389^ KI mice (Figure 3a,b). Table 1 directly compares the magnitude of LPS responses between the two genotypes.

In addition, this LPS treatment increased the percentage of ACSA-2^+^ astrocytes in WT mice, as previously reported [31], but not in GSK3β Ser^389^ KI mice (Figure 3c). Regarding brain infiltration of immune cells, LPS caused an accumulation of neutrophils in WT mice but had no effect in GSK3β Ser^389^ KI mice (Figure 3d). Finally, LPS administration did not promote lymphocyte accumulation in the brain in either WT or GSK3β Ser^389^ KI mice (Figure 3e). 

Altogether, these observations indicate that the inactivation of GSK3β by Ser^389^ phosphorylation is essential to prevent a tonic inflammatory state in the brain but contributes to acute brain inflammation in response to stimuli such as LPS.

### 2.4. GSK3β Ser^389^ Phosphorylation Affects Glial Activation in the Hippocampus

**Figure 4:**
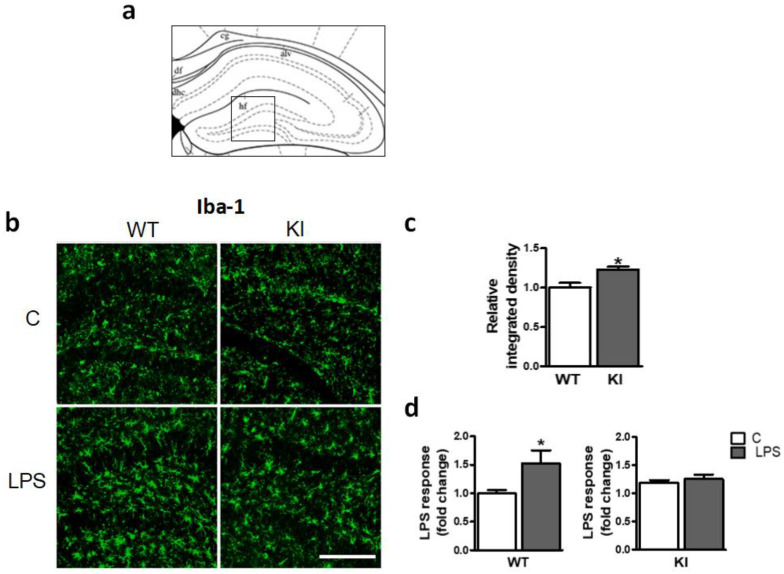
Gliosis in response to LPS is altered in GSK3β Ser^389^ KI mice. Adult WT and GSK3β Ser^389^ KI mice were injected intraperitoneally with LPS or vehicle (C). After 3 days, brains were processed for fluorescent microscopy. (**a**) A coronal hippocampus section adapted from the mouse brain atlas [45] indicates the localization of the images, which include the dentate gyrus. (**b**) Brain sections were immunolabeled for microglia/macrophage markers Iba-1. Graphs depict the integrated density of Iba-1 immunoreactivity, which is a measurement of glial reactivity that reflects both the intensity and the extension of glial staining. (**c**) Comparison of control WT and KI animals. (**d**) LPS response in each mouse genotype. All values are expressed as increase respect to WT controls treated with vehicle (mean ± S.E.M., *n* = 4–5). Student *t*-test was applied. *, *p* < 0.05. Scale bar, 100 µm. WT, wild-type; KI, GSK3β Ser^389^ knockin; C, vehicle; LPS, lipopolysaccharide.

Next, we examined the possible influence of hippocampal GSK3β Ser^389^ phosphorylation on basal glial activation and the glial response to inflammatory stimuli. For this aim, Iba-1 labeling was evaluated by microscopy in the dentate gyrus (Figure 4a). First, WT mice were evaluated. Micrographs reveal low basal glial activation, but a robust increase in glial immunoreactivity in response to LPS injection (0.5 mg/kg, 3 days) (Figure 4b). Quantitation of the integrated density for Iba-1 immunostaining showed values that were 53% higher in WT mice after treatment (Figure 4d). These changes were statistically significant and are consistent with the prominent cellular hypertrophy caused by LPS in the mouse hippocampus previously reported [43,44].

The analysis of the hippocampus of Ser^389^ KI mice revealed differences in basal microglial activation with respect to WT mice. Thus, although visual inspection of micrographs showed a moderate glial activation in mutant animals (Figure 4b), integrated density measurements indicated that the increase in glial immunoreactivity was statistically significant (Figure 4c). Interestingly, Ser^389^ KI mice showed no signs of further activation after LPS treatment (Figure 4b), and quantitative analysis confirmed that LPS was unable to increase Iba-1 immunostaining in the hippocampus (Figure 4d), suggesting that glial cell activation by LPS is impaired in the absence of GSK3β Ser^389^ phosphorylation.

### 2.5. Peripheral Immune Response to LPS Was Not Affected in GSK3β Ser^389^ KI Mice

**Figure 5:**
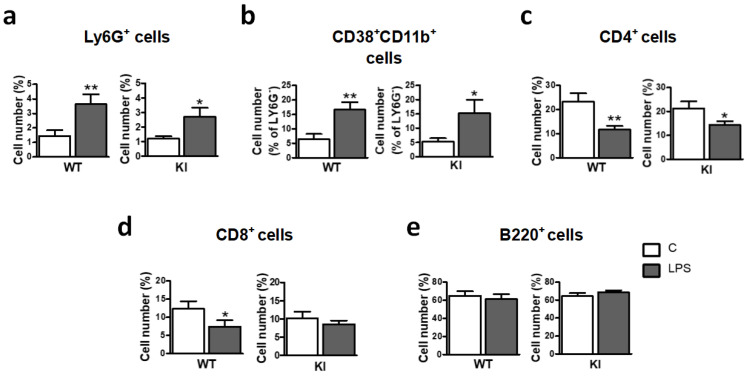
Similar peripheral immune response to LPS in GSK3β Ser^389^ KI and WT mice. Adult WT and GSK3β Ser^389^ KI mice were i.p. injected with LPS. After 3 days, spleen cells were extracted and processed to evaluate, by flow cytometry, the number of (**a**) Ly6G^+^ neutrophils, (**b**) CD38^+^CD11b^+^ monocytes/macrophages, (**c**) CD8^+^, (**d**) CD4^+^, and (**e**) B220^+^ cells. Histograms show the effects of LPS on cell number, including WT and KI mice. Student t-test was applied to evaluate the significance of changes induced by LPS in each genotype. Mean ± S.E.M is shown (*n* = 5–7). *, *p* < 0.05; **, *p* < 0.01. WT, wild-type; KI, GSK3β Ser^389^ knockin; i.p., intraperitoneal; C, vehicle; LPS, lipopolysaccharide.

Our previous experiments unveil the profound consequences of GSK3β inactivation by Ser^389^ phosphorylation on the brain response to LPS. Next, we addressed whether similar effects are present in systemic immunity. Since LPS is known to cause activation of spleen innate immune cells [46], spleen cell populations from both WT and GSK3β Ser^389^ KI mice were evaluated 3 days after LPS injection (0.5 mg/kg). the number of Ly6G^+^ neutrophils (Figure 5a) and CD38^++^CD11b^++^Ly6G^−^ monocytes/macrophages (Figure 5b) significantly increased in WT mice in response to LPS treatment. These LPS effects, expected in WT mice, were also observed in GSK3β Ser^389^ KI mice (Figure 5a,b). No differences between genotypes were obtained either when lymphocyte populations from the spleen were analyzed, as the percentages of spleen CD4^+^ cells (Figure 5c) and CD8^+^ cells (Figure 5d) were reduced by LPS also in both mice strands (the reduction in mutant mice was statistically significant only for CD4^+^ cells); and B220^+^ B cells were unaffected by LPS independently of genotype (Figure 5e). These observations are in contrast to the differential effects of LPS found in the brain of both genotypes. Thus, while GSK3β Ser^389^ KI mice are resistant to the neuroinflammation triggered by LPS, these mice are not systemic desensitized to LPS. Therefore, the effects of GSK3β Ser^389^ phosphorylation seem to be selective to the brain.

### 2.6. LPS-Induced NF-κB Pathway Activation in the Hippocampus Is Abolished in GSK3β Ser^389^ KI Mice

**Figure 6:**
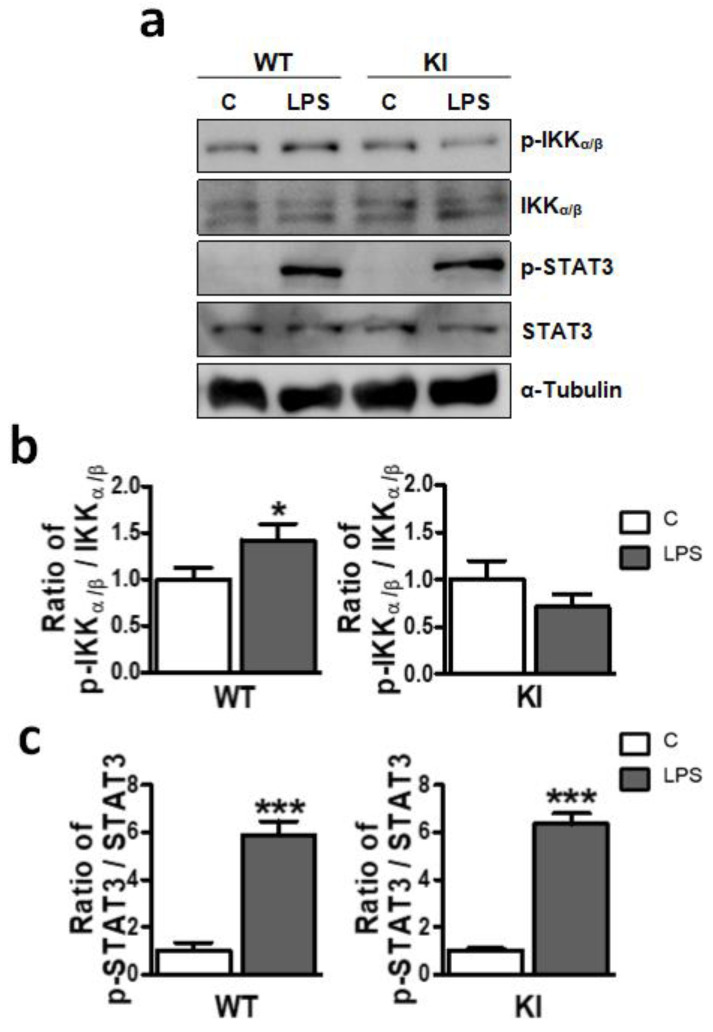
Failure to inactivate GSK3β by Ser^389^ phosphorylation differently affects NF-κB and STAT3 signaling in LPS-treated mice. Activation of the NF-κB and STAT3 pathways were examined in whole-cell lysates from hippocampus 4 h after i.p. injection of LPS (0.5 mg/kg) or vehicle (C) in both WT and GSK3β Ser^389^ KI mice. Blots illustrate the Western blot analysis of phospho-IKKα/β, total IKKα/β, phospho-STAT3, and total STAT3. (**a**) α-tubulin detection was included as a loading control. Associated graphs correspond to (**b**) phospho-IKKα/β/IKKα/β and (**c**) phospho-STAT3/STAT3 ratios, obtained from the densitometric analysis of Western blot bands. Student t-test was applied to compare untreated controls (C) and LPS-treated animals in each genotype (mean ± S.E.M, *n* = 5–6). *, *p* < 0.05; ***, *p* < 0.001. Data are representative of four independent experiments for pIKK/IKK and three independent experiments for pSTAT3/STAT3. WT, wild-type; KI, GSK3β Ser^389^ knockin; i.p., intraperitoneal; C, vehicle; LPS, lipopolysaccharide.

NF-κB is a transcriptional factor essential for LPS to induce inflammation [47]. In addition, NF-κB has been shown to be regulated by GSK3β in the brain [2,19,23]. We therefore examined NF-κB activation in the brain in response to LPS by the analysis of IKK phosphorylation, an early step in the NF-kB activation pathway. WT and GSK3β Ser^389^ KI mice were i.p. administered with LPS (0.5 mg/kg) and after 4 h, the hippocampus was harvested. Whole-cell extracts were used to examine phospho-IKKα/β and total IKKα/β by Western blotting. As expected, LPS increased IKK phosphorylation in the hippocampus in WT mice (Figure 6a,b). In contrast, LPS failed to induce IKK phosphorylation in GSK3β Ser^389^ KI mice (Figure 6a,b).

Activation of STAT3 has also been associated with LPS-induced inflammation [48]. We therefore examined the phosphorylation of STAT3 by Western blot analysis. In contrast to NF-kB activation, phosphorylation of STAT3 was strongly induced in the hippocampus of both WT and GSK3β Ser^389^ KI mice in response to LPS (Figure 6a,c). Thus, preventing GSK3β inactivation by Ser^389^ phosphorylation selectively impairs NF-κB signaling, which is consistent with a role for GSK3β as an upstream regulator of the NF-κB pathway in the hippocampus. These results suggest that the GSK3β pathway influences neuroinflammation by controlling key inflammatory mediators, such as NF-κB.

## 3. Discussion

In the brain, dysregulation of GSK3β activity is linked to multiple neuropathologies in which chronic inflammation is a common hallmark [25]. Since a large literature supports that inhibition of GSK3β by Ser^9^ phosphorylation upregulates anti-inflammatory mediators and downregulates pro-inflammatory cytokines [23,28,30], the mechanism commonly proposed to explain defective GSK3β inactivation in neuroinflammatory pathologies is the failure to phosphorylate Ser^9^ [3]. However, unusually high levels of phospho-Ser^389^ GSK3β have been detected in the brain [4], suggesting that this alternative pathway for GSK3β inactivation may also play a prominent role within the brain. The experiments presented here uncover the implication of GSK3β Ser^389^ phosphorylation in neuroinflammation, which adds to previous evidence suggesting the involvement of this pathway in brain DNA repair and neuronal survival [6,7].

One of the key findings in the present work is that the brain of GSK3β Ser^389^ KI mice shows sustained gliosis and increased immune cell infiltration, which is consistent with the notion that preventing GSK3β inactivation through Ser^389^ phosphorylation leads to neuroinflammation. Data from our flow cytometry experiments clearly point to increased neuroinflammation in these animals, as the number of activated microglia/macrophages, ACSA 2^+^ astrocytes, and infiltrated neutrophils in the brain of Ser^389^ GSK3β KI mice were significantly higher. However, despite micro-astrogliosis being clearly observed by flow cytometry, analysis of hippocampus micrographs from brain sections of Ser^389^ GSK3β KI mice revealed that hypertrophy of microglia/macrophages was only mild. Similar results were observed when other brain areas were analyzed to assess the extent and intensity of gliosis (not shown). Therefore, we conclude that, in the absence of pro-inflammatory stimuli, the lack of GSK3β Ser^389^ phosphorylation leads to a degree of neuroinflammation that should be classified as low-grade. 

Numerous studies support the association between GSK3 hyperactivity and neurodegeneration in neurological disorders [25]. In this context, the inflammatory response that we observed in Ser^389^ GSK3β KI mice could be the consequence of neurodegeneration, as previous studies have detected neuronal death in the cortex and hippocampus of these animals [6]. This is in accordance with the observation that DNA damage inactivates GSK3β through Ser^389^ phosphorylation as a mechanism to prevent cell death and promote DSB repair [7,8]. In consequence, the brain of KI mice, in which Ser^389^ phosphorylation is prevented, is not protected against DSB-induced neuronal death. The link between phospho-Ser^389^ GSK3β expression and cell survival was first proposed in lymphocytes, as a mechanism implicated in the adaptive immune response. Thus, the absence of GSK3β Ser^389^ phosphorylation in KI mice was found to induce necroptosis in B cells during its late maturation, which is a highly inflammatory type of cell death related to several pathologies [9]. It is also interesting to note that not only Ser^389^ phosphorylation but also Ser^9^ phosphorylation can be triggered to inactivate GSK3β in response to DNA damage. However, Ser^389^ GSK3β KI mice present a neurodegenerative phenotype [6] not present in Ser^9^ GSK3β KI mice [49,50], indicating that Ser^389^ phosphorylation plays specific roles in the control of GSK3β activity and that there are scenarios in which Ser^9^ phosphorylation cannot compensate for the loss of Ser^389^ phosphorylation. Accordingly, the intriguing link between Ser^389^ GSK3β deregulation, neuroinflammation, and neurodegeneration unveiled here needs to be further explored to clarify whether altered GSK3β phosphorylation at Ser^389^ may be a key factor in neuropathologies in which chronic inflammation is distinctive, such as Alzheimer’s disease. 

In addition, the mild but persistent activation of neuroimmune cells observed in GSK3β Ser^389^ KI mice is consistent with the hypothesis that failure to phosphorylate Ser^389^ may be associated with immunosenescence. This is an age-related process characterized by a decline in immune functions together with an increase in the expression of pro-inflammatory mediators; which leads to chronic, low-grade inflammation [1,51]. Several authors have pointed to the involvement of GSK3 deregulation in age-related inflammation [1,52]. It has also been proposed that the abnormal function of microglia observed in the context of brain immunosenescence may eventually cause neurodegeneration [53]. Thus, accelerated immunosenescence in Ser^389^ GSK3β KI mice could contribute to the loss of neurons previously reported in these animals [6]. 

LPS has been extensively used to induce inflammation in both “in vivo” and “in vitro” models. However, there is evidence that the dose of LPS administered largely determines the type of inflammatory response induced [54,55,56]. High doses of LPS produce septic shock-like responses; while moderate LPS doses trigger a persistent low-grade inflammation closer to that observed in many chronic diseases [42]. In this context, it is important to emphasize that the LPS dose selected for our experiments (0.5 mg/kg, i.p.) is moderate, and is far from the LD50 reported for LPS in mice (10–25 mg/kg) [57,58]. In agreement with previous reports [40,59], we found that such an LPS dose is not lethal and is sufficient to increase gliosis and brain infiltration of neutrophils in WT animals, while lymphocyte infiltration is not affected. Remarkably, such moderate (but significant) LPS response was abolished in the brain of KI mice with impaired inactivation of GSK3β by Ser^389^ phosphorylation. According to this observation, extended GSK3β hyper activation in the brain may eventually downregulate the sensitivity to pro-inflammatory insults. It is noteworthy that the defective response to LPS in the mutant mice is compatible with the mechanism of immunotolerance or resistance, a common process in the CNS aimed at reducing glial cell activation and limiting brain damage from excessive inflammation [60,61]. In agreement with this idea, a previous study showed that GSK3 activation in mice can induce immunotolerance and suppress LPS responses [62]. 

It is also interesting to consider that the brain effects described in this study show high specificity. Thus, contrarily to the brain, most spleen cell populations evaluated in WT and Ser^389^ GSK3β KI animals presented the same profile in unstimulated conditions, as well as a similar response to LPS. This suggests that the GSK3β inactivation pathway dependent on Ser^389^ phosphorylation plays specific roles in the regulation of neuroinflammation. In agreement with this hypothesis, brain cells show a high expression of phospho-Ser^389^ GSK3β compared to other tissues, and this expression is tightly regulated during brain development [5]. 

Finally, according to our findings, the regulation of GSK3β by phosphorylation at Ser^389^ is specifically mediated by the NF-kB signaling pathway, since STAT3 activation was unaffected in Ser^389^ GSK3β KI. Many downstream inflammatory mediators are regulated by NF-kB activation, including cytokines, receptors, transcription factors and enzymes. Identifying which of these NF-kB targets are influenced by Ser^389^ GSK3β phosphorylation is beyond the scope of our work, but it will be an exciting goal for future studies aiming to clarify the mechanisms of neuroinflammation. 

## 4. Materials and Methods

### 4.1. Animals

C57BL/6J Wild-type (WT) mice were purchased from Jackson Laboratories. C57BL/6 GSK3β Ser^389^ KI mice, in which the Ser^389^ residue of GSK3β was replaced by Ala to prevent phosphorylation, were generated as previously described [8]. All mice were housed in the animal facility associated with the Albacete Medical School under a 12 h light/dark cycle. Food and water were provided ad libitum. Four to six-month-old adult mice were used for the experiments.

### 4.2. LPS Administration

To induce an inflammatory response, mice received a single intraperitoneal (i.p.) injection of 0.5 mg/kg LPS. Salmonella enterica LPS (serotype typhimurium, L7261 Sigma-Aldrich, Madrid, Spain) was dissolved in sterile, endotoxin-free 0.9% saline at 10 mg/mL. Three days after treatment, mice were euthanized, and the brain and spleen were removed for immunohistochemistry or flow cytometry analysis of glial cells and infiltrated immune cells. For Western blotting analysis, animals were euthanized 4 h after i.p. injection of 0.5 mg/kg LPS, as LPS has previously been reported to upregulate inflammatory proteins in mouse and rat brains 2–4 h after i.p. administration [63,64]. 

### 4.3. Flow Cytometry

For flow cytometry analysis of brain tissue and spleen cells, animals were anesthetized with an i.p. injection of tribromoethanol, which has been recommended for acute terminal studies. The low dose of tribromoethanol used (200 mg/kg) provides light anesthesia without serious adverse effects or influence on inflammatory cytokine expression [65]. Animal procedures were performed after verifying the absence of reflexes.

#### 4.3.1. Brain Tissue Dissociation and Percoll Gradient Isolation of Neural Cells

Mice were intracardially perfused with 50 mL of 0.9% NaCl before tissue collection to remove circulating cells from brain blood vessels. After perfusion, the brains were removed, the meninges and choroid plexus were carefully separated, and the cerebellum and olfactory bulbs were discarded. The remaining tissue was placed in cold Hank’s Balanced Salt Solution, which is calcium chloride and magnesium chloride free (HBSS [-]CaCl2/[-]MgCl2: Gibco, Madrid, Spain, 14175). Tissue samples were then finely chopped with a scalpel before digestion.

Chopped tissue was then placed in MACS C tubes (Miltenyi Biotec, Pozuelo de Alarcón, Spain, 130-093-237) in a total volume of 5 mL, and processed with a mechanic dissociator at 6 rpm for 30 min. Enzymatic dissociation was performed according to our own protocol [31] using papain (100 U; Worthington, LK003178) and dispase II (6 U; Sigma-Aldrich, Madrid, Spain, D4963) in Earle’s Balanced Salt Solution (EBSS [+]CaCl2/[+]MgSO4; Gibco, Madrid, Spain, 24010). DNAse I (100 U; Sigma-Aldrich, Madrid, Spain, DN25) was added to the solution to remove the DNA mucus that adversely affects cell viability [66]. Prior to use, papain was activated for 30 min at 37 °C in 5% CO2. To stop digestion, samples were diluted with cold HBSS and placed on ice. This solution was then pipetted 10 times using 5 mL pipettes and filtered through a 70 μm cell strainer (BD, 352350). The resulting single-cell suspension was centrifuged at 300× *g* for 10 min at RT.

A 30% Percoll^TM^ (GE Healthcare, Madrid, Spain, 17-0891-01) gradient was used to remove myelin and enrich the homogenate into viable glial cells. For this purpose, a stock solution of isotonic Percoll (SIP) was prepared (9:1 Percoll in 10× HBSS [-]CaCl2/[-]MgCl2; Gibco, 14185), and cell pellets were resuspended in 30% (*v*/*v*) SIP (in 1× HBSS). The 30% Percoll gradient was carried out by adding 3 mL of SIP to 7 mL of 1× HBSS containing the cell suspension in a 14 mL polypropylene tube. Each gradient was centrifuged without a brake at 300× *g* for 30 min at 18 °C. After centrifugation, the cells from the bottom layer were collected, washed with 1× HBSS, centrifugated for 10 min at 300× *g*, and resuspended in 10 mL of 1× red blood cell lysis buffer at 4 °C. After lysis, cell suspension was centrifuged at 300× *g* for 10 min, and the resulting cell pellet was finally resuspended in 50 μL of blocking buffer before staining for flow cytometry. 

#### 4.3.2. Spleen Cell Dissociation

Spleens were placed on a Petri dish containing RPMI 1640 and cut into small pieces. The resulting tissue was passed into a 70 μm cell strainer (BD, 352350) and broken up in a circular motion with the ribbed top of a syringe’s plunger. RPMI 1640 was then passed through the strainer. The disintegrated tissue was transferred to 50 mL tubes (Sarstedt, Barcelona, Spain, 62.547.254) and centrifuged at 100 g for 10 min. The supernatant containing the cell suspension was then collected and centrifuged again at 300 g for 10 min. The resulting supernatant was removed, and the pellet was resuspended in 10 mL phosphate-buffered saline (PBS) with 1% FBS to count the number of cells under the microscope, after which it was re-centrifuged at 300 g for 5 min. Only 500,000 cells per sample were used. Each sample was then resuspended in 50 µL of blocking buffer before staining for flow cytometry.

#### 4.3.3. Extracellular Flow Cytometry Staining

Flow cytometry staining was performed as previously described [31]. To block non-specific staining of surface antigens, which is essential for the detection of microglia/macrophage antigens, 0.5 μL of mouse FcR Blocking Reagent (1:100 dilution; Miltenyi Biotec, Pozuelo de Alarcón, Spain, 130-059-901) was added to blocking buffer containing the cell suspension and incubated for 10 min. Subsequently, the mix containing fluorochrome-conjugated antibodies was added (Table 2). Cells were kept at 4 °C in the dark, for 15 min; and then washed with 1 mL of blocking buffer and centrifugated for 5 min at 300× *g*. Finally, the cell pellets were resuspended in 500 μL of PBS and samples were immediately used for flow cytometry. Data were acquired using a MACSQuant flow cytometer and analyzed with MACSQuantify software (Miltenyi Biotec). Prior to sample analysis, fluorescent calibration beads (1000 beads/µL) were added to count the absolute number of cells. After excluding cellular debris and aggregates, a minimum of 10,000 events corresponding to individual viable cells were examined in each analysis. The cell number measurements are presented as normalized with respect to their controls. 

### 4.4. Immunohistochemistry

Mice were deeply anesthetized and intracardially perfused with saline 0.9% (*w*/*v*). Their brains were collected and post-fixed with 4% paraformaldehyde for 24 h. After post-fixation, they were cryoprotected with 30% sucrose and frozen at −20 °C until sectioning. Tissue sections (20 μm) were cut using a cryostat (ThermoFisher, Microm™ HM550), thaw-mounted onto Superfrost Plus slides (ThermoFisher, J1800AMNZ) and finally stored at −20 °C until staining.

Immunohistochemical staining of tissue sections was performed as described before [67]. Primary and secondary antibody solutions were diluted in 1× PBS with 1% BSA. The sections were incubated with the primary antibody anti-rabbit Iba-1 (1:500 dilution; Wako Chemicals, Barcelona, Spain, 019-19741). Alexa Fluor 488-conjugated goat anti-rabbit IgG (1:2000 dilution; ThermoFisher, Madrid, Spain, A11031) was used as secondary antibody. Appropriate positive control tissue was included, as well as primary and secondary negative controls. Fluorescent labeling images were acquired using a ZEISS Apotome.2 microscope with an axiocam 503 mono digital camera and the Zen Blue control software version 2.3 (Carl Zeiss). A 10× objective (Plan-APOCHROMAT, V10×, NA 0.45, AIR) was used to visualize the hippocampus. The integrated density within the regions of interest was calculated with ImageJ software. This parameter corresponds to the sum of the intensity values for each pixel in the region selected. Therefore, it is used for quantitation of the number of cells stained in combination with the staining intensity of each cell. 

### 4.5. Western Blot Analysis

Mouse brains were quickly removed, and the hippocampi were immediately dissected and frozen. Tissue was prepared using a hypertonic lysis buffer containing protease inhibitors as previously described [68]. The primary antibodies used were anti-rabbit phospho-IKKα/β (1:1000 dilution; Cell Signaling, Alcobendas, Spain, 2861), anti-rabbit IKK α/β (1:1000 dilution; Santa Cruz Biotechnology, Heidelberg, Germany, sc-7607), anti-rabbit phospho-STAT3 (1:1000 dilution; Cell Signaling, 9145) and anti-mouse STAT3 (1:1000 dilution; Santa Cruz Biotechnology, sc-8019). Anti-mouse α-tubulin (1:2000 dilution; Santa Cruz Biotechnology, sc-32293) was used as loading control. Anti-rabbit HRP (1:5000 dilution; Jackson ImmunoResearch Laboratories, Baltimore, USA, 111-035-144) and anti-mouse HRP (1:5000 dilution; Jackson ImmunoResearch Laboratories, 115-035-003) were used as secondary antibodies. Protein band quantification was carried out by band densitometry analysis with the NIH ImageJ Software (Version 1.50i, https://imagej.nih.gov/ij/, accessed on 1 March 2022). To ensure equal loads, the integrated density obtained for each band was expressed as a relative value with respect to the appropriate housekeeping loading control.

### 4.6. Statistical Analysis

Results were statistically analyzed with the GraphPad Prism software (San Diego, CA, USA). Data are presented as mean ± standard error of the mean (S.E.M) of generally four independent experiments (*n* = 4–9). After confirming the normal distribution of data by the Shapiro-Wilk test, statistical significance between two groups was determined by the Student *t*-test for parametric data. Differences were considered statistically significant when probability (*p*) values were less than 0.05 (*), 0.01 (**), or 0.001 (***). Figures show representative experiments.

## 5. Conclusions

In summary, our findings indicate that failure to inactivate GSK3β by Ser^389^ phosphorylation leads to chronic inflammation and endotoxin tolerance and that the inhibition of GSK3β by Ser^9^ and Ser^389^ phosphorylation plays complementary and not redundant roles in the neuroimmune response. Therefore, the specific consequences of GSK3β Ser^389^ phosphorylation for neuropathology and brain aging should be investigated.

## Figures and Tables

**Table 1:** Differences in LPS effects between WT and GSK3β Ser^389^ KI mice. Values represent cell number after LPS treatment in each genotype and are expressed as an increase with respect to vehicle-treated controls (mean ± S.E.M). Asterisks indicate statistically significant differences between the effects of LPS in WT and GSK3β Ser^389^ KI animals. WT, wild-type; KI, GSK3β Ser^389^ knockin; LPS, lipopolysaccharide. *, *p* < 0.05; **, *p* < 0.01.

	LPS Response (Fold Change)		
	WT	KI	Statistical Significance
CD11b^++^CD45^high^ activated microglia/macrophages	1.67 ± 0.15	0.83 ± 0.12	**
CD11b^+^CD45^low^ resting microglia	0.76 ± 0.085	1.14 ± 0.08	**
ACSA2^+^ astrocytes	1.37 ± 0.14	0.91 ± 0.16	*
Ly6G^+^ neutrophils	3.12 ± 0.80	1.04 ± 0.20	**
CD11b^−^ lymphocytes	1.26 ± 0.40	0.74 ± 0.15	No

**Table 2:** Antibodies and reagents used for flow cytometry analysis.

Antibody/ Reagent	Clone	Cell Targets	Concentration	Company (Reference)
ACSA-2-APC	IH3-18A3	Neonatal and adult Astrocytes	1:25	Miltenyi Biotec (130-117-535)
B220 (CD45R)	RA3-6B2	B lymphocytes	1:25	Miltenyi Biotec (130-102-259)
Calibration beads			1000 beads/mL	Miltenyi Biotec (130-093-607)
CD11b-PEVio770	REA592	Macrophages, microglia, granulocytes, NK cells, and subsets of dendritic cells	1:50	Miltenyi Biotec (130-113-246)
CD4	REA604	T helper cells, regulatory T cells	1:25	Miltenyi Biotec (130-119-132)
CD8	REA601	Cytotoxic T cells	1:25	Miltenyi Biotec (130-102-490)
CD38-APCVio770	REA616	Subsets of macrophages, microglia, B cells and T cells	1:10	Miltenyi Biotec (130-109-337)
CD45-PE	30F11	Hematopoietic cells except for erythrocytes	1:25	Miltenyi Biotec (130-117-498)
FcR Blocking Reagent			1:100	Miltenyi Biotec (130-092-575)
LY6G-Vioblue	1A8	Neutrophils	1:10	Miltenyi Biotec (130-110-449)

## Data Availability

All data are contained within the manuscript.

## Supplementary Materials

The following supporting information can be downloaded at https://www.mdpi.com/article/10.3390/ijms24010337/s1.

## Abbreviations

CNS	Central Nervous System
EBSS	Earle’s Balanced Salt Solution
FSC	Forward-scattered light
GSK3	Glycogen Synthase Kinase 3
HBSS	Hank’s Balanced Salt Solution
IKK	IkB kinase complex
i.p.	Intraperitoneal injection
KI	Knock-in
LPS	Lipopolysaccharide
NF- kB	Nuclear factor kB
PBS	Phosphate-buffered saline
S.E.M	Standard error of the mean
SIP	Stock solution of isotonic Percoll
SSC	Side-scattered light
STAT3	Signal transducer and activator of transcription 3
TLR	Toll-like receptor
WT	Wild type
