# Supplementary material for: GSK3β Inhibition by Phosphorylation at Ser389 Controls Neuroinflammation
Source: Int J Mol Sci. 2022 Dec 25;24(1):337. doi: 10.3390/ijms24010337 (PMC9820301; doi:10.3390/ijms24010337)
Supplement: Supplementary file 1 [file ijms-24-00337-s001.zip › Figure S1.pdf]

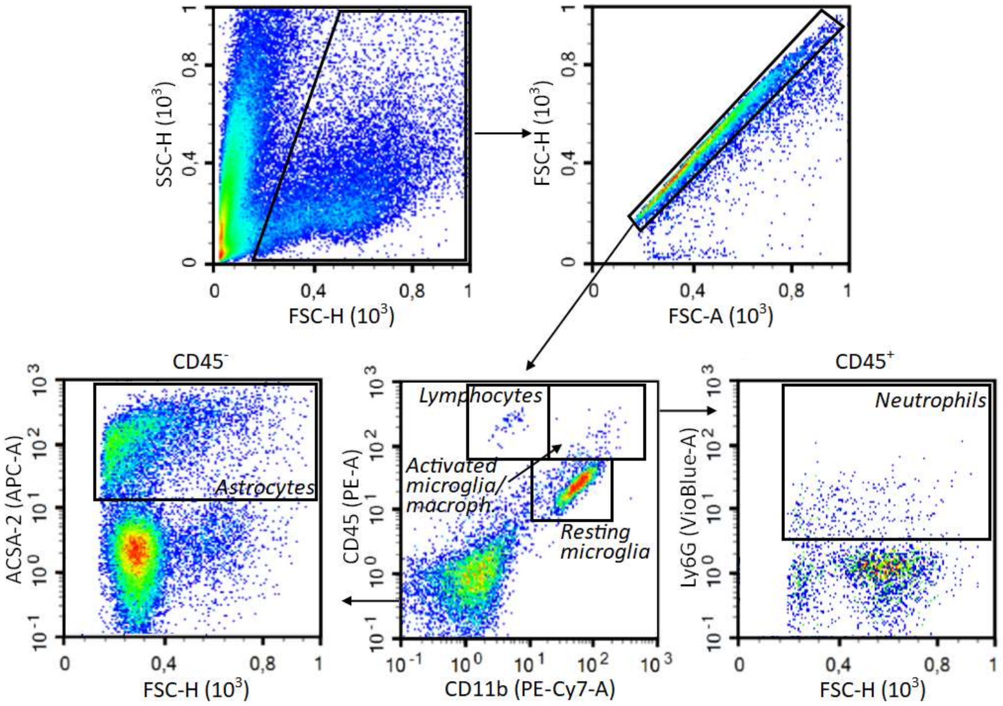

Figure S1. Gating strategy for flow cytometry analysis of brain cells implicated in neuroimmunity. Tissue was mechanically and enzymatically dissociated to obtain intact single cells from adult brain. Representative density plots from WT animals show gating for $CD11b^{++}CD45^{high}$ activated microglia/macrophages, $CD11b^{++}CD45^{low}$ resting microglia, $CD11b^{-}$ lymphocytes, $Ly6G^{+}$ neutrophils and $ACSA\text{-}2^{+}$ astrocytes.
