# Supplementary material for: GSK3β Inhibition by Phosphorylation at Ser389 Controls Neuroinflammation
Source: Int J Mol Sci. 2022 Dec 25;24(1):337. doi: 10.3390/ijms24010337 (PMC9820301; doi:10.3390/ijms24010337)
Supplement: Supplementary file 1 [file ijms-24-00337-s001.zip › Figure S2.pdf]

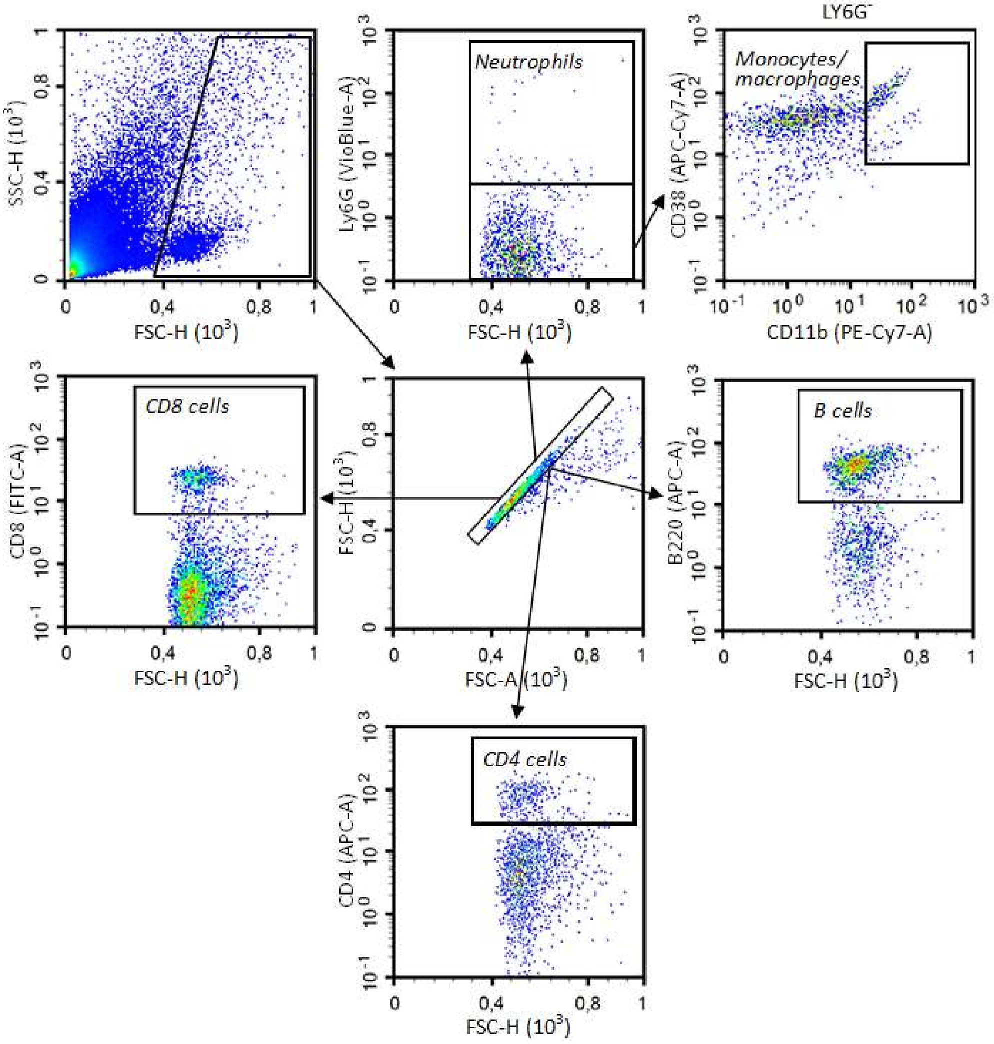

Figure S2. Gating strategy for flow cytometry analysis of spleen cells. After mechanical dissociation, spleen cells were processed for flow cytometry. Subpopulations of $Ly6G^+$ neutrophils, $Ly6G^-$ $CD38^{++}CD11b^+$ monocytes/macrophages, as well as $CD8^+$, $CD4^+$ and $B220^+$ cells were gated for anal- ysis. Representative density plots are shown.
